# Conducting Polymer-Infused Electrospun Fibre Mat Modified by POEGMA Brushes as Antifouling Biointerface

**DOI:** 10.3390/bios12121143

**Published:** 2022-12-07

**Authors:** Jesna Ashraf, Sandy Lau, Alireza Akbarinejad, Clive W. Evans, David E. Williams, David Barker, Jadranka Travas-Sejdic

**Affiliations:** 1 Polymer Biointerface Centre, School of Chemical Sciences, The University of Auckland, Auckland 1010, New Zealand; 2 The MacDiarmid Institute of Advanced Materials and Nanotechnology, Wellington 6140, New Zealand; 3 Hub for Extracellular Vesicles Investigation (HEVI), Department of Obstetrics and Gynecology, The University of Auckland, Auckland 1010, New Zealand; 4 School of Biological Sciences, The University of Auckland, Auckland 1010, New Zealand

**Keywords:** electrospinning, conducting polymer, antifouling, proteins, BSA, POEGMA brush, cell viability and proliferation

## Abstract

Biofouling on surfaces, caused by the assimilation of proteins, peptides, lipids and microorganisms, leads to contamination, deterioration and failure of biomedical devices and causes implants rejection. To address these issues, various antifouling strategies have been extensively studied, including polyethylene glycol-based polymer brushes. Conducting polymers-based biointerfaces have emerged as advanced surfaces for interfacing biological tissues and organs with electronics. Antifouling of such biointerfaces is a challenge. In this study, we fabricated electrospun fibre mats from sulphonated polystyrene-block-poly(ethylene-*ran*-butylene)-block-polystyrene (sSEBS), infused with conducting polymer poly(3,4-ethylenedioxythiophene) (PEDOT) (sSEBS-PEDOT), to produce a conductive (2.06 ± 0.1 S/cm), highly porous, fibre mat that can be used as a biointerface in bioelectronic applications. To afford antifouling, here the poly(oligo (ethylene glycol) methyl ether methacrylate) (POEGMA) brushes were grafted onto the sSEBS-PEDOT conducting fibre mats via surface-initiated atom transfer radical polymerization technique (SI-ATRP). For that, a copolymer of EDOT and an EDOT derivative with SI-ATRP initiating sites, 3,4-ethylenedioxythiophene) methyl 2-bromopropanoate (EDOTBr), was firstly electropolymerized on the sSEBS-PEDOT fibre mat to provide sSEBS-PEDOT/P(EDOT*-co-*EDOTBr). The POEGMA brushes were grafted from the sSEBS-PEDOT/P(EDOT*-co-*EDOTBr) and the polymerization kinetics confirmed the successful growth of the brushes. Fibre mats with 10-mers and 30-mers POEGMA brushes were studied for antifouling using a BCA protein assay. The mats with 30-mers grafted brushes exhibited excellent antifouling efficiency, ~82% of proteins repelled, compared to the pristine sSEBS-PEDOT fibre mat. The grafted fibre mats exhibited cell viability >80%, comparable to the standard cell culture plate controls. Such conducting, porous biointerfaces with POEGMA grafted brushes are suitable for applications in various biomedical devices, including biosensors, liquid biopsy, wound healing substrates and drug delivery systems.

## 1. Introduction

Surface fouling caused by the adhesion of proteins, peptides, lipids and microorganisms is a major challenge that limits the performance of various medical devices, including diagnostic and biosensing platforms [1], tissue engineering constructs [2], and blood-contacting devices [3]. The interactions pertaining to the nonspecific adsorption of proteins include hydrophobic effects, van der Waals and coulumbic forces [4,5]. For example, biointerfaces for the capture and release of biological targets, such as circulating cancer cells (CTCs) [6] and extracellular vesicles (EVs) [7], are affected by protein fouling which results in obstruction of capture probes and contamination by proteins [8]. It is therefore important to develop biointerfaces with antifouling properties to suppress the non-specific adsorptions of proteins and other biomaterials. A number of antifouling functionalisations have been developed, such as peptide-based antifouling chemistries [9], surface modifications with zwitterionic polymers and poly(ethylene glycol) (PEG) brushes [10,11,12,13]. PEG-based polymer brushes are considered as one of the most promising classes of antifouling materials and have been extensively studied especially in the biomedical field [14,15]. The excellent antifouling properties of the surfaces grafted by the PEG polymer brushes were attributed to high surface coverage by the brushes, charge-neutrality of PEGs, protective water barrier properties, lack of hydrogen bond donating sites and the steric repulsion exhibited by long PEG chains [16,17,18]. For instance, Mousavi et al. developed packaging for a biomedical implant based on medical-grade poly(dimethyl siloxane) (PDMS) [19]. To prevent protein and cell adhesion, POEGMA brushes were grafted onto PDMS, increasing the hydrophilicity and protein fouling resistance of the PDMS surface. In another study, Venault et al. found that the generation of network-like PEGMA structures on expanded polytetrafluoroethylene (ePTFE) membranes, prepared by the atmospheric-plasma induced PEGylation process, has substantially increased the wettability of the resulting membranes. The antifouling PEGylated ePTFE membranes showed high resistance towards the adsorption of fibrinogen and suppressed the adhesion of Gram-negative and Gram-positive bacteria [20].

Surface-initiated atom transfer radical polymerization (SI-ATRP) technique has been extensively implemented as an efficient polymerization procedure for growing polymer brushes from surfaces through a surface-initiated controlled polymerisation [21]. It affords end-functionalised brushes with low dispersity. The SI-ATRP works with a wide range of functionalised monomers [22,23,24]. An example is a work by Rodda et al., where POEGMA brushes were grafted via SI-ATRP from benzyl chloride initiator on polystyrene surfaces. This platform finds applications as low fouling scaffolds for tissue engineering [25]. Another study by Albers et al., demonstrated Cu^0^-mediated SI-ATRP for growing structured POEGMA brushes on multidimensional material surfaces [26].

Conducting polymers have gained interest as biosensors [27] and biointerfaces [28] owing to their biocompatibility, both ionic and electronic conductivity, and processibility [29]. Because of their ability to interact with cells and biomolecules, conducting polymers have been utilized as biointerfaces that record neural activities [30], for cell stimulations [31], platforms for electrochemical biosensors [32] and in drug delivery systems [33]. Among the conducting polymers, PEDOT has been extensively studied owing to its biocompatibility, environmental stability, excellent conductivity, electroactivity in water and well-defined electrochemical properties [34,35]. Previous work from our group utilized polystyrene-block-poly(ethylene-*ran*-butylene)-block-polystyrene (sSEBS) fibre mats coated with PEDOT as a biointerface for the capture and release of breast cancer cell line EVs [7], but the effect of proteins fouling on those substrates has not been investigated. 

The current work focuses on enhancing the antifouling properties of such substrates to suppress the non-specific adsorption of proteins. To that end, a copolymer of EDOT and ethylenedioxythiophene-(3,4-ethylenedioxy thiophene) methyl 2-bromopropanoate) (EDOTBr) was electrodeposited onto the sSEBS-PEDOT fibre mat, followed by grafting of hydrophilic POEGMA brushes using SI-ATRP. The POEGMA brush-grafted fibre mats showed significantly reduced protein adsorption (repelled >80% proteins) compared to the pristine sSEBS-PEDOT. Furthermore, we demonstrated that human microvascular endothelial cells (HMEC-1) are viable on the POEGMA brush-grafted fibre mats, comparable to the standard cell culture plate. Hence, these protein-resistant substrates are promising as biointerface for applications in biomedical devices. 

## 2. Materials and Methods

3,4-Ethylenedioxythiophene (EDOT) and (2,3-dihydrothieno [3,4-b][1,4]dioxin-2-yl)methanol were obtained from AK scientific, oligoethylene glycol methyl ether methacrylate (OEGMA), 2-bromopropionyl bromide, copper bromide, 2,2′-bipyridine and lithium perchlorate were purchased from Sigma-Aldrich (Auckland, New Zealand). Dichloromethane, acetonitrile, sodium bicarbonate, magnesium sulphate, ethanol and methanol were provided by ECP Ltd. (Auckland, New Zealand). Triethylamine (TEA) was purchased from BDH (Poole, UK). Bovine serum albumin (BSA) was acquired from pH Scientific (Auckland, New Zealand). Pierce^TM^ Bicinchoninic acid (BCA) Protein Assay Kit was purchased from ThermoFisher Scientific (Auckland, New Zealand). Iron (III) chloride was obtained from ECP Ltd. and all the deionised water was obtained from MilliQ (18.2 MΩ cm) system (Arium Pro Ultrapure Water Systems, Sartorius, Germany). 

### 2.1. Synthesis of (3,4-Ethylenedioxythiophene) Methyl 2-Bromopropanoate (EDOTBr)

Synthesis of EDOTBr was carried out following a procedure reported earlier [36]. To a solution of dichloromethane (6 mL), (2,3-dihydrothieno [3,4-b][1,4]dioxin-2-yl)methanol (1.16 mmol, 200 mg), 4(dimethylamino)pyridine (0.871 mmol, 106 mg) and trimethylamine (TEA) (1.51 mmol, 210 μL) was added dropwise 2-bromopropionyl bromide (1.96 mmol, 243 μL) under nitrogen and the mixture was stirred at 0 °C. The reaction was then continued with stirring at room temperature for 3 h, washed with brine (1 × 20 mL), 10 mM NaHCO_3_ (1 × 20 mL) and deionized water (2 × 20 mL) and dried over anhydrous MgSO_4_. Excess solvent was removed under vacuum and the residue was purified by silica gel column chromatography (hexane/ethyl acetate 3:1), affording monomer EDOTBr as a pale-yellow oil. ^1^H NMR (400 MHz, CDCl3): d 1.83 (3H, d, CH3), 4.04–4.47 (6H, m), 6.35 (2H, s, Ar) (Figure S5).

### 2.2. Sulfonation of Polystyrene-Block-Poly(Ethylene-Ran-Butylene)-Block-Polystyrene (SEBS)

The SEBS block copolymer (10 gm) was dissolved in chloroform (100 mL) and the solution was heated to 40 °C. To this solution, sulfuric acid (0.8 mL, 0.015 mol) and acetic anhydride (2.533 mL, 0.027 mol) were added, and the mixture was stirred at 40 °C for 5 h. Triethylamine (TEA, 4 mL) was added dropwise into the above mixture to terminate the reaction by neutralization. Excess solvent (chloroform) was removed by rotary evaporation and the solid yellow polymer obtained was washed with ethanol in a Soxhlet extractor for 24 h to remove the remaining TEA and salts. The washed polymer was then dried under vacuum for 24 h at 40 °C.

### 2.3. Fabrication of Electrospun Fibre Mats

A polymeric solution of sSEBS/PEO (2/0.25 gm) in 20 mL chloroform was drown into a glass syringe (5 mL) with a metallic needle. The electrospinning box temperature was set to 30 °C and maintained under nitrogen flow to maintain the optimum level of humidity (less than 10%). The collector plate was a stainless steel (20 × 20 cm) square plate and is kept around 15 cm apart from the needle tip which was grounded. This needle tip was connected at the positive terminal to the DC voltage supply. Pumping (5 mL/h) was started after setting the voltage supply to 18 kV. The needle tip was cleaned every 10 min to remove the solidified polymer and the spinning continued until the polymer solution in the syringe was used up. The pump and voltage supply were switched off every time while cleaning the needle tip. The fibre mat on the collector plate was then dried under vacuum at 40 °C overnight, followed by removing of the fibre mat from the plate.

### 2.4. Chemical Oxidative Polymerisation of EDOT on the Electrospun Fibre Mats

The fibre mats were cut into sizes of 6 × 11 cm, the pieces were dipped into a 20 mL solution of 0.5 M EDOT in methanol and kept in a Petri dish at room temperature for 24 h. A flat, rectangular-shaped container was filled with 1.5 M aqueous iron chloride solution. The dried EDOT-soaked mat was stretched over the opening of the container lid and sealed on top of it. The container was then placed upside down so that the mat was fully covered by the solution and was kept in an oven at 60 °C for 3 h. After 3 h, the mat was removed from the container, rinsed with methanol and then soaked in methanol overnight. The rinsing and soaking were repeated 3 times with fresh methanol before the mat was dried under ambient conditions to obtain PEDOT-coated conducting electrospun fibre mats (sSEBS-PEDOT).

### 2.5. Electropolymerisation of P(EDOT-co-EDOTBr) on the sSEBS-PEDOT Fibre Mats

Electrocopolymerization of EDOT and EDOTBr was performed with a CH Instruments potentiostat using a three-electrode electrochemical cell. The sSEBS-PEDOT fibre mat was used as the working electrode, a platinum mesh and Ag/AgCl were used as counter and reference electrodes, respectively. A solution of 0.1 M LiClO4 in acetonitrile was used as the electrolyte. Electropolymerisation was performed potentiodynamically in the potential range from −0.5 V to 1.5 V, with 10 cycles at a scan rate of 100 mV/s. The obtained sSEBS-PEDOT/P(EDOT*-co-*EDOT-Br) fibre mats were then washed with acetonitrile and deionized water before drying under nitrogen.

### 2.6. Grafting POEGMA Brushes on the sSEBS-PEDOT/P(EDOT-EDOTBr) Fibre Mat

The SI-ATRP was performed based on a previously reported method [37] to graft POEGMA brushes on the surface of the sSEBS-PEDOT/P(EDOT*-co-*EDOT-Br) fibre mats, using initiating sites on EDOTBr mer. In a Schlenk tube, sSEBS-PEDOT-P(EDOT*-co-*EDOT-Br) fibre mats, catalyst (CuBr, 72 mg) and the ligand (2,2′-bypyridine, 340 mg) were added and the tube was sealed, followed by continuous evacuation and N_2_ infusion. A solution of OEGMA (1 mmol) and isopropanol (4 mL) was added to a round bottom flask, sealed with septa and purged with N_2_ for 1 h. The monomer solution was then added to the Schlenk tube using a syringe. The reaction continued for 3 h, after which the grafted fibre mat (sSEBS-P(EDOT*-co-*EDOTBr)*-g-*POEGMA) was taken out and washed several times with ethanol and deionised water to remove any residues on the surface and dried overnight under vacuum.

### 2.7. Protein Adsorption Test

Protein adsorption tests were conducted by bicinchoninic acid assay (Pierce™ BCA Protein Assay Kit; ThermoFisher Scientific). Bovine serum albumin (BSA) was used as a model protein to study the protein adhesion on the fibre mats. The POEGMA brush-grafted electrospun fibre mats (5 × 8 mm) were incubated in 500 µL solution of BSA in PBS (200 µg/mL) for 2 h at room temperature (22 °C). The fibre mats were rinsed thoroughly in PBS prior to the incubation. The amount of adsorbed protein was calculated by subtracting the concentration of solution after incubation from the initial concentration read. The unknown concentrations were elucidated from the calibration curve prepared from BSA solutions (prepared according to the assay procedure) of various concentration and their corresponding absorbance at 562 nm. Three repeats were measured and the average value was taken

### 2.8. Cell Viability and Proliferation

Human microvascular endothelial cells (HMEC-1) were seeded at a concentration of 2000 cells/well into Nunc™ MicroWell™ 96-Well inlaid with either grafted POEGMA fibre mats, or directly onto the cell culture plate surface. Cells were cultured in MDCB medium containing 10% fetal bovine serum and 1% penicillin/streptomycin, under 5% CO_2,_ 21% O_2_ and at 37 °C. After 24 h of culture, cell viability was assessed. The culture medium containing 10% PrestoBlue™ cell viability reagent (Thermofisher, Auckland, New Zealand, catalogue number A13261) was added to each well of a 96 well plate and incubated at 37 °C for 1 h. The medium was then removed from the wells and transferred to a new 96-well plate for fluorescence measurement with 530/25 nm excitation and 590/35 emission filter sets using a Synergy™ 2 microplate reader (BioTek, Winooski, VT, USA). The fluorescence reading was corrected with blank wells containing only the medium containing PrestoBlue™ Cell Viability Reagent. PrestoBlue™ was used as a metabolic indicator comparable to MTT (3-(4,5-dimethylthiazol-2-yl)-2,5-diphenyltetrazolium bromide) and Alamar blue assays for cellular viability and proliferation of HMEC-1 cells [38] (immortalized cell line derived from human dermal endothelial cells).

### 2.9. Scanning Electron Microscopy (SEM)

SEM images were obtained using an FEI Phillips XL30 S-FEG instrument (Amsterdam, the Netherlands) connected with an EDAX Phoenix, Pleasanton, CA, USA (Energy Dispersive Spectroscopy) EDS detector.

### 2.10. Four-Point Probe Conductivity Meter

The conductivity of sSEBS fibre mats, PEDOT-sSEBS and grafted fibre mats were calculated from the sheet resistance obtained using the JANDEL four-point probe conductivity meter with a 1 mm pins’ spacing. The thickness of the fibre mats was measured using the Mitutoyo multifunction dial gauge.

### 2.11. Raman Spectroscopy

Raman spectra of the bare sSEBS electrospun fibre mat and the PEDOT-coated conducting fibre mat were recorded using the Renishaw Raman system 1000 spectrometer with a light source of 532 nm with 1% laser power and an acquisition time of 30 s. Raman data were analysed using LabSpec software.

### 2.12. X-ray Photoelectron Spectroscopy (XPS)

XPS analysis was performed using Kratos Axis UltraDLD spectrometer equipped with an Al Ka X-ray radiation source (hν = 1486.69 eV) operating at 150 W and a hemispherical electron energy analyzer. During data collection, a pressure range of 10^−9^ Torr was maintained in the analysis chamber. The spectra were analysed using CasaXPS software.

### 2.13. ^1^H NMR Measurement

^1^H NMR spectra of SI-ATRP reaction solution at different time intervals were recorded using a Bruker Advance III 400 MHz instrument in D_2_O (Billerica, MA, USA). Ethanol was used as the internal standard and the conversion of monomer was calculated by comparing the integrated values of monomer peaks (6.40, 6.48) with the internal standard at various time intervals.

### 2.14. Water Contact Angle Measurement

Contact angle measurements on the sSEBS, sSEBS-PEDOT, sSEBS-PEDOT/P(EDOT*-co-*EDOTBr) and grafted fibre mats were conducted using a CAM 100 contact angle meter (KSV Instruments, Helsinki, Finland).

## 3. Results and Discussion

### 3.1. Fabrication of POEGMA Brush Grafted Electrospun Fibre Mat

**Figure 1:**
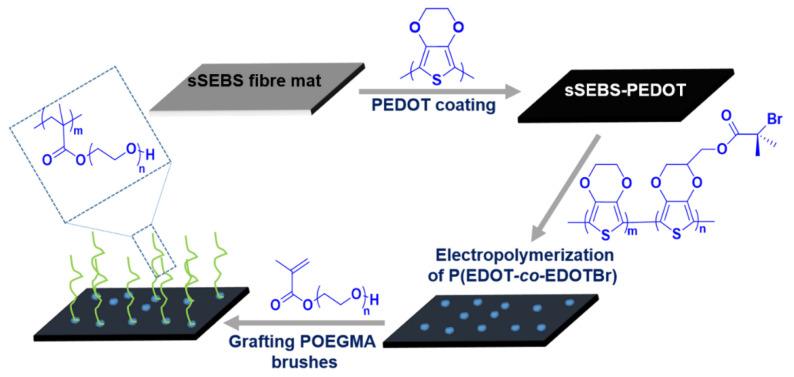
Schematic representation of fabrication of sSEBS-PEDOT/P(EDOT-co-EDOTBr) fibre mat with grafted POEGMA brushes.

The POEGMA brush-grafted sSEBS-PEDOT/P(EDOT*-co-*EDOTBr) fibre mats were prepared as outlined in Figure 1. The electrospun sSEBS fibre mats were prepared by electrospinning a polymer solution of sulfonated polystyrene-*block*-poly(ethylene-*ran*-butylene)-*block*-polystyrene (sSEBS) and poly(ethylene oxide) (PEO) in chloroform. The sSEBS fibre mat was then converted into a conducting fibre mat by chemical oxidative polymerization of EDOT in the presence of iron (III) chloride as the oxidizing agent to form PEDOT-coated conducting electrospun (sSEBS-PEDOT) fibre mat, as published by us earlier [7]. A copolymer of EDOT (10 mM) and EDOTBr (1 mM) was then electropolymerized onto the sSEBS-PEDOT fibre mat to form sSEBS-PEDOT/P(EDOT*-co-*EDOTBr) fibre mat. The copolymer is the macroinitiator for an SI-ATRP of POEGMA. The EDOT in the ((EDOT*-co-*EDOTBr) copolymer acts as a spacer between the EDOTBr macroinitiator units to control the grafting density. The sSEBS-PEDOT/P(EDOT*-co-*EDOTBr) substrate was then used for SI-ATRP of hydrophilic POEGMA brushes. During SI-ATRP, the macroinitiator P(EDOT-EDOTBr) initiates the polymerization reaction by creating radicals and transfer of a halogen atom to the catalyst ligand complex which is oxidized thereafter. During the propagation, these radicals attack the OEGMA monomer units and create intermediate radical species. OEGMA monomers with 9 ethylene glycol units were used. The reaction between the intermediate radical species and transition metal catalyst-ligand complex resulted in the termination of the reaction forming the grafted polymer on the fibre surface.

### 3.2. Characterization of Electrospun Conducting sSEBS-PEDOT Fibre Mat

**Figure 2:**
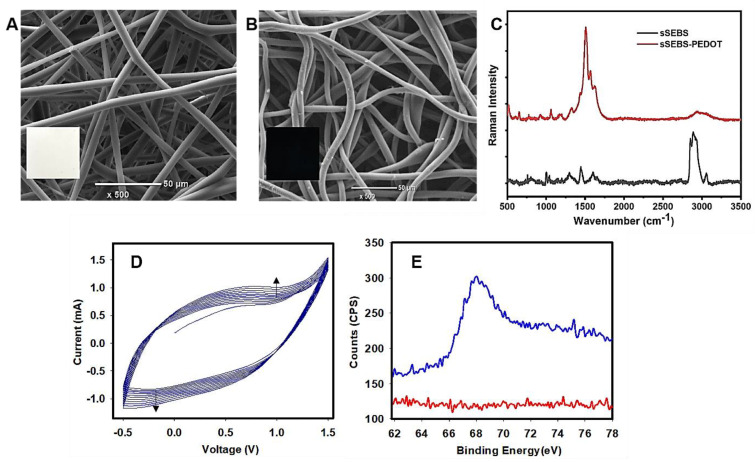
SEM images of (**A**) an electrospun sSEBS fibre mat (inset: photographs of sSEBS fibre mat), (**B**) PEDOT coated conducting electrospun fibre mat (sSEBS-PEDOT) (inset: photographs of sSEBS-PEDOT fibre mat), (**C**) Raman spectra of pristine and PEDOT coated electrospun sSEBS fibre mat, (**D**) Cyclic voltammograms of the electrodeposition of P(EDOT*-co-*EDOTBr) (10:1 mol:mol) onto sSEBS-PEDOT in the potential range of -0.5 V to +1.5 V at 100 mV/s for 10 cycles, in 0.1 M LiClO_4_ in acetonitrile, and (**E**) XPS spectra of Br 3d of sSEBS-PEDOT fibre mat (red) and sSEBS-PEDOT/P(EDOT*-co-*EDOTBr) fibre mat (blue).

As reported earlier [7], the PEDOT coating was visually confirmed by the colour change of the fibre mat from white to dark blue (insets, Figure 2A,B) which is attributed to the reaction between the oxidant and the EDOT monomer. Successful incorporation of PEDOT into the sSEBS fibre mats was confirmed by the SEM images and Raman spectroscopy. The SEM images of sSEBS fibre mat (Figure 2A) showed a smooth surface, while the sSEBS-PEDOT fibre mats (Figure 2B) exhibited a rough fibre surface morphology indicating the presence of PEDOT. The increase in the mean fibre diameter of the sSEBS-PEDOT (8.92 ± 1.3 µm) compared to that of sSEBS fibres (6.66 ± 1.2 µm) confirmed further the successful incorporation of PEDOT into the sSEBS fibre mat. Furthermore, sSEBS-PEDOT fibre mats exhibited an electrical conductivity of 2.06 ± 0.1 S/cm due to the presence of conducting PEDOT. Raman spectroscopy was performed to investigate the chemical composition of fibre mats after the incorporation of PEDOT into the sSEBS fibre mats. The distinctive C-H stretching band [24] at 2900 cm^−1^, along with a few weak bands at 3051 cm^−1^, 1597 cm^−1^, 1445 cm^−1^, 1296 cm^−1^ and 1008 cm^−1^ of sSEBS substrate are in sharp contrast to the Raman spectra of PEDOT (Figure 2C) which displays the characteristic bands at 1425 cm^−1^ of symmetric C_α_ = C_β_ stretching and 990 cm^−1^ corresponding to the oxyethylene ring deformation [39]. The other bands in the Raman spectrum of sSEBS-PEDOT at 700 cm^−1^ of symmetric C-S-C stretching, 1100 cm^−1^ assigned to C-O-C deformation, 1266 cm^−1^ of C_α_-C_α_ inter-ring stretching and 1366 cm^−1^ of C_β_-C_β_ stretching, further affirm the PEDOT polymerisation [26] on the sSEBS fibre mat.

### 3.3. Electrochemical Copolymerisation of P(EDOT-co-EDOTBr) on sSEBS-PEDOT Fibre Mat

The electrocopolymerisation of EDOT and EDOTBr (10 mM EDOT: 1 mM EDOTBr) was carried out potentiodynamically in 0.1 M LiClO_4_ in acetonitrile, within a potential window of −0.5 V to +1.5 V at 100 mV/sec scan rate. The copolymerization voltammograms (Figure 2D) showed an increase in the redox currents at high potentials with the increasing number of cycles, indicating successful deposition of conducting P(EDOT*-co-*EDOTBr). Electrochemical homopolymerisations and the copolymerisation of EDOT and EDOTBr have been studied earlier by us [36]; however, not on sSEBS-PEDOT electrospun fibre mat. Here, the onset of copolymerisation for P(EDOT*-co-*EDOTBr) occurs at a potential of +1.2 V. A broad oxidation peak was observed in the voltammograms at +0.6 V (vs. Ag/AgCl) with a corresponding reduction peak at –0.3 V (vs. Ag/AgCl). To confirm the successful incorporation of the macroinitiator, EDOTBr into P(EDOT*-co-*EDOTBr), the mat surfaces were characterized by XPS analysis. The high-resolution XPS scan of Br 3d confirmed the successful deposition of the EDOTBr into the P(EDOT*-co-*EDOTBr). The Br 3d peak was completely absent in the sSEBS-PEDOT while it was evident for the electrodeposited fibre mat (Figure 2E). The high-magnification SEM images of sSEBS-PEDOT and sSEBS-PEDOT/P(EDOT*-co-*EDOTBr) are shown in Figure S1. The SEM-EDS analysis over a 12 mm^2^ mapping area of the sSEBS-PEDOT fibre mat and sSEBS-PEDOT/P(EDOT*-co-*EDOTBr) fibre mat verified the copolymerisation of P(EDOT*-co-*EDOTBr). The fibre mats with electrodeposited P(EDOT*-co-*EDOTBr) showed 2.68 weight % of bromine on the surface, while the sSEBS-PEDOT fibre mats did not show any traces of bromine (Figure S2), confirming the presence of ATRP initiating sites on the copolymerised P(EDOT*-co-*EDOTBr).

### 3.4. Grafting POEGMA Brushes from sSEBS-PEDOT/P(EDOT-co-EDOTBr) Fibre Mat

**Figure 3:**
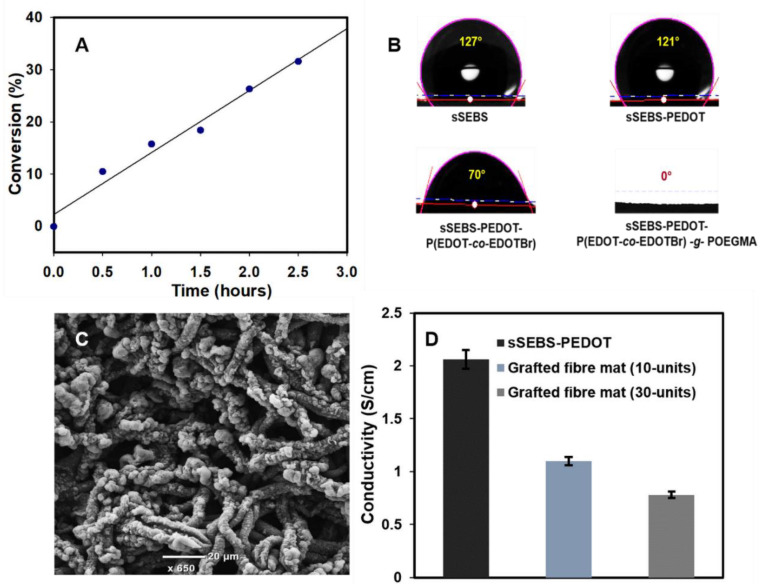
(**A**) SI-ATRP kinetics graph for POEGMA grafting on electrospun fibre mats, (**B**) Contact angle measurement of sSEBS, sSEBS-PEDOT, sSEBS-PEDOT/P(EDOT*-co-*EDOTBr) and sSEBS-PEDOT/P(EDOT*-co-*EDOTBr)*-g-*POEGMA (30-mers) fibre mat, (**C**) SEM image of sSEBS-PEDOT/P(EDOT*-co-*EDOTBr)*-g-*POEGMA (30-mers) fibre mat, and (**D**) Conductivity of the sSEBS-PEDOT and sSEBS-PEDOT/P(EDOT*-co-*EDOTBr)*-g-*POEGMA fibre mat with 10- and 30-mers graft length (*n* = 5).

SI-ATRP technique was utilized to graft POEGMA brushes on the sSEBS-PEDOT/P(EDOT*-co-*EDOTBr) fibre mat. The NMR spectra of SI-ATRP reaction solution at different time intervals revealed the conversion of OEGMA monomer to POEGMA polymer (Figure S3). The molecular weight of the monomer is multiplied with the conversion (at each time interval) to give the chain length which corresponds to the number of grafted units. The decrease in the peak intensity of the monomer peaks was used to study the kinetics of the reaction. The kinetics graph showing the conversion of OEGMA monomer at different time intervals of SI-ATRP (Figure 3A) indicates the increased chain length as the grafting proceeds. An optimum level of polymer brush surface coverage and chain length are significant for a surface to exhibit superior antifouling properties [40]. Here, we studied the antifouling properties of the mats grafted with two different POEGMA chain lengths: 10- and 30-mer units. The 30-mer long polymer graft was obtained after 2.5 h of grafting, while 10-mer long polymer graft was obtained by quenching the reaction at 1 hr of the reaction time. The water contact angle of the sSEBS-PEDOT/P(EDOT*-co-*EDOTBr)*-g-*POEGMA fibre mat surface was recorded at each step of functionalisation. As displayed in Figure 3B, the sSEBS and sSEBS-PEDOT fibre mats exhibited a highly hydrophobic surface with contact angles of 127 ± 0.7° and 121 ± 0.5°, respectively. The macroinitiator deposited sSEBS-PEDOT/P(EDOT*-co-*EDOTBr) surface showed a much lower contact angle of 70 ± 1.2°. The grafted sSEBS-PEDOT/P(EDOT*-co-*EDOTBr)*-g-*POEGMA) fibre mat exhibited a completely hydrophilic surface with 0° contact angle (Figure 3B), in accordance to the literature where zero contact angle for the PEGMA grafted polysulfone membranes was reported [41]. The SEM images of 30-mer POEGMA brush grafted fibre mats are shown in Figure 3C, further confirming the successful grafting of POEGMA brushes. The bundles of long-chain POEGMA brushes look like being coiled into mushroom-like structures and present a very different morphology to the fibre mats of sSEBS-PEDOT (Figure 3C). A recent study on PVDF/styrene-*co*-maleic anhydride-*g*-PEG flat membranes showed similar surface morphology for the PEG brush grafted membrane [42]. Figure 3D shows the electrical conductivity of the POEGMA brush grafted fibre mats and the sSEBS-PEDOT fibre mats. The conductivity of grafted fibre mats with 10-mers POEGMA brush (1.1 ± 0.05 S/cm) and 30-mers (0.78 ± 0.04 S/cm) is somewhat lower compared to that of sSEBS-PEDOT (2.06 ± 0.1 S/cm). The decrease in electrical conductivity is attributed to the presence of the insulating POEGMA brush on the conducting fibre mat. However, a conductivity of 0.78 ± 0.04 S/cm is still appreciable and likely sufficient in bioelectronics and similar applications [43,44], including a release of biological targets as per our previous work.

### 3.5. Antifouling Properties of sSEBS-PEDOT/P(EDOT-co-EDOTBr)-g-POEGMA Fibre Mat

The antifouling properties of sSEBS-PEDOT/P(EDOT*-co-*EDOTBr)*-g-*POEGMA and sSEBS-PEDOT (as control) fibre mats were studied using BCA protein kit assay [45]. A spectrophotometer (Nanodrop, Implen NP80 nanophotometer, Germany) was used to measure the absorbance of the Bovine Serum Albumin (BSA) solutions before and after the incubation of fibres in BSA solutions. A calibration curve was obtained for standard BSA solutions (Figure S4) and used to ascertain the number of BSA proteins adsorbed on the surfaces from the difference in concentration of protein solutions before and after fibre mat incubation. The higher the protein concentration in the solution after incubation, the lesser the protein adsorbed on the fibre mat.

**Figure 4:**
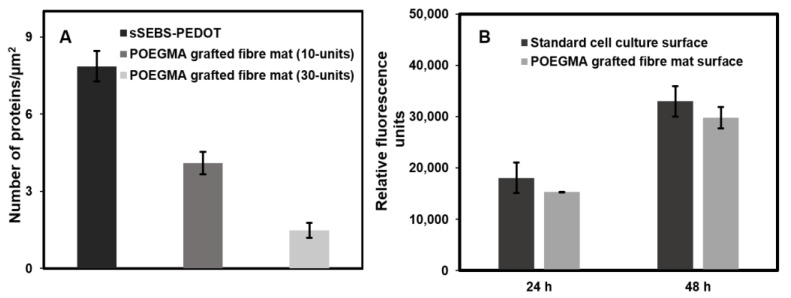
(**A**) The number of BSA proteins adsorbed on the grafted sSEBS-PEDOT/P(EDOT-*co*-EDOTBr)-*g*-POEGMA with 10- and 30-mers graft lengths and the sSEBS-PEDOT fibre mat, surfaces, after 2 h incubation in a solution of BSA in PBS (200 µg/mL); calculated as number per µm^2^ of the projected area (n = 3). (**B**) The fluorescent signals obtained from the cells grown on the standard cell culture plate and POEGMA grafted fibre mat surface.

The number of protein particles adsorbed on the fibre mat surfaces was calculated by estimating the total projected area of the fibre mat, solution concentration before and after fibre incubation and the volume of each incubation solution. The number of BSA molecules adsorbed on the POEGMA brush grafted fibre mat surface (graft length of 30-units) was 82% lower than on the ungrafted sSEBS-PEDOT fibre mat surface, as shown in Figure 4A. Such good fouling resistance is attributed to the hydrophilicity and the energy barrier created by the conformational entropy of the POEGMA brushes [19,46]. The surface hydration layer formed by the interaction between water and the POEGMA chains creates a steric hindrance against proteins approaching the surface [47]. Furthermore, the results showed a correlation between the protein resistance and graft length of the POEGMA chains. The grafted fibre mat with 10-mers graft length only had 4.1 ± 0.6 BSA proteins per µm^2^ of the projected area compared to the pristine fibre mat with 7.86 ± 0.44 protein molecules per µm^2^. The longer 30-mer POEGMA brush grafted fibre mat showed as low as 1.47 ± 0.3 proteins per µm^2^ of the projected area (82% decrease compared to the pristine sSEBS-PEDOT mat), indicating excellent fouling resistance. Hence, the results suggest that the POEGMA brush grafted electrospun fibre mat, sSEBS-PEDOT/P(EDOT-*co*-EDOTBr)-*g*-POEGMA, can be potentially employed as an effective antifouling, electrically addressable biointerfaces. Examples of several antifouling modifications on different electrode surfaces are listed in Table S1.

### 3.6. Cell Viability and Proliferation Assessment Using PrestoBlue^TM^

To study the cell viability and proliferation of cells, human microvascular endothelial cells, HMEC-1, were seeded into Nunc™MicroWell™ 96-Well inlaid with either POEGMA grafted fibre mats, or directly onto the cell culture plate surface. After 24 h of culture, cell viability was assessed and the culture medium containing 10% PrestoBlue™ cell viability reagent was added to each well of a 96 well plate and incubated at 37 °C for 1 h. The fluorescence measurements were taken to obtain the level of cell viability. The fluorescence signal obtained from cells cultured using POEGMA grafted fibre mats as a growth surface was not significantly different to that from cells grown on the standard plasma-treated plastic cell culture surface at both 24 h after seeding (mean ± SD, 15,322 ± 2493 RFU vs. 18,062 ± 62 RFU, *n* = 3, *p* = 0.10) and at 48 h (29,811 ± 2985 RFU, vs. 32,987 ± 2064 RFU, *n* = 3, *p* = 0.20). The fluorescence intensity increased by approximately 80% at 48 h following the seeding of the cells onto the POEGMA mats compared to 24 h, suggesting the proliferation of the cells during the culture. The assessment of the cell’s viability and proliferation on the functionalized POEGMA brush surface suggests that the generated substrates do not induce cytotoxicity, nor do they inhibit proliferation in vitro of HMEC-1 cells. This finding is consistent with recent publications assessing cellular viability on POEGMA surfaces [48,49,50]. Whilst, the viability of HMEC-1 cells grown on the POEGMA surface was not significantly different to the plasma-treated standard cell culture plastic surface, the average viability was slightly lower, but still above 80%, and the mat is considered non-cytotoxic according to the ISO10993 standard for in vitro cytotoxicity. These results are attributed to the presence of the biocompatible conducting polymer PEDOT and the subsequent grafting of the biocompatible POEGMA brushes on the surface of the fibre mat.

## 4. Conclusions

We have successfully grafted hydrophilic POEGMA brushes on conducting polymer-coated electrospun fibre mats that exhibited excellent antifouling properties to BSA. With the increase in the POEGMA graft length, the antifouling performance of the grafted fibre mats improved significantly (over 80% reduction in BSA fouling), while the fibre mat retained a good electrical conductivity in the range of 1–3 S/cm. Moreover, the POEGMA brush-grafted fibre mat showed nontoxicity to HMEC-1 cells with high cell viability (>80%) indicating a proliferation index of ~0.8–1. These substrates are suitable to be employed as electroactive and conductive biointerfaces in, for example, electrochemical biosensors, capture/release platforms, devices for disease diagnostics and drug delivery systems.

## Data Availability

Not applicable.

## Supplementary Materials

The following supporting information can be downloaded at: https://www.mdpi.com/article/10.3390/bios12121143/s1, Figure S1. SEM images of sSEBS-PEDOT (left) and sSEBS-PEDOT/P(EDOT-co-EDOTBr) (right); Figure S2. SEM-EDS analysis of bromine in (A) SSEBS-PEDOT fibre mat and (B) electropolymerized fibre mat bearing the brominated macroinitiator [SSEBS-PEDOT-P (EDOT-co-EDOTBr)]; Figure S3. NMR spectra of OEGMA monomer from the ATRP reaction solution at different time intervals; Figure S4. Calibration curve of BSA standards at different concentration vs. absorbance measured using BCA protein assay; Figure S5. 1H NMR spectrum of synthesized EDOTBr; Table S1. Examples of antifouling modifications on different electrode surfaces [51,52,53,54,55].
