# Supplementary material for: Conducting Polymer-Infused Electrospun Fibre Mat Modified by POEGMA Brushes as Antifouling Biointerface
Source: Biosensors (Basel). 2022 Dec 7;12(12):1143. doi: 10.3390/bios12121143 (PMC9775683; doi:10.3390/bios12121143)
Supplement: Supplementary file 1 [file biosensors-12-01143-s001.zip › biosensors-2008733-supplementary.pdf]

biosensors

Supporting Information

Conducting Polymer Infused Electrospun Fibre Mat Modified by POEGMA Brushes as Antifouling Biointerface

*Jesna Ashraf, Sandy Lau, Alireza Akbarinejad, Clive W. Evans, David E. Williams, David Barker, Jadranka Travas-Sejdic**

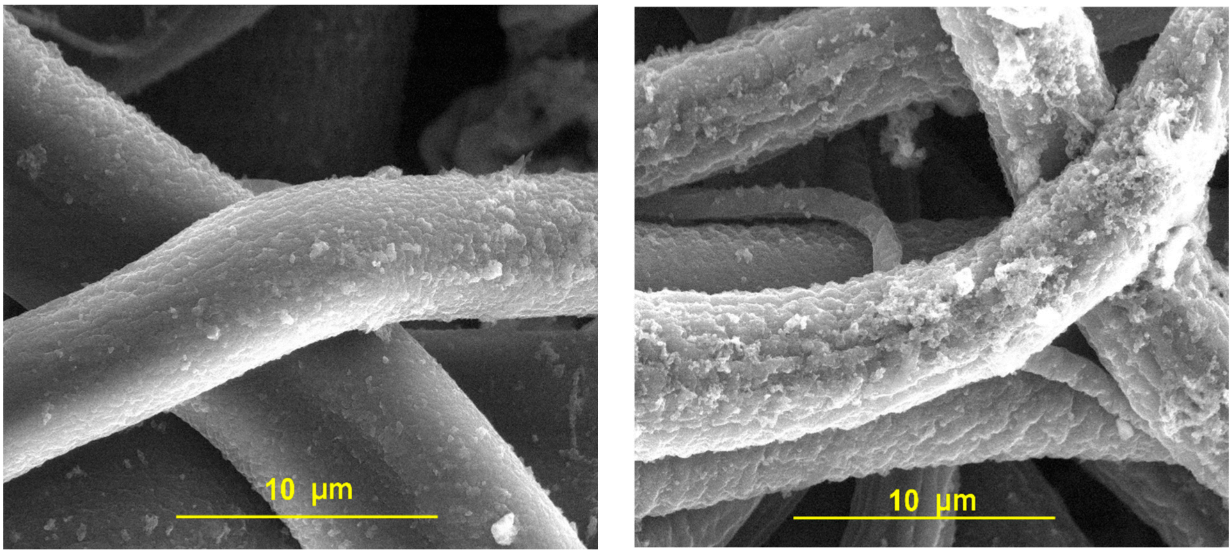

Figure S1. SEM images of sSEBS-PEDOT (left) and sSEBS-PEDOT/P(EDOT-co-EDOTBr) (right).

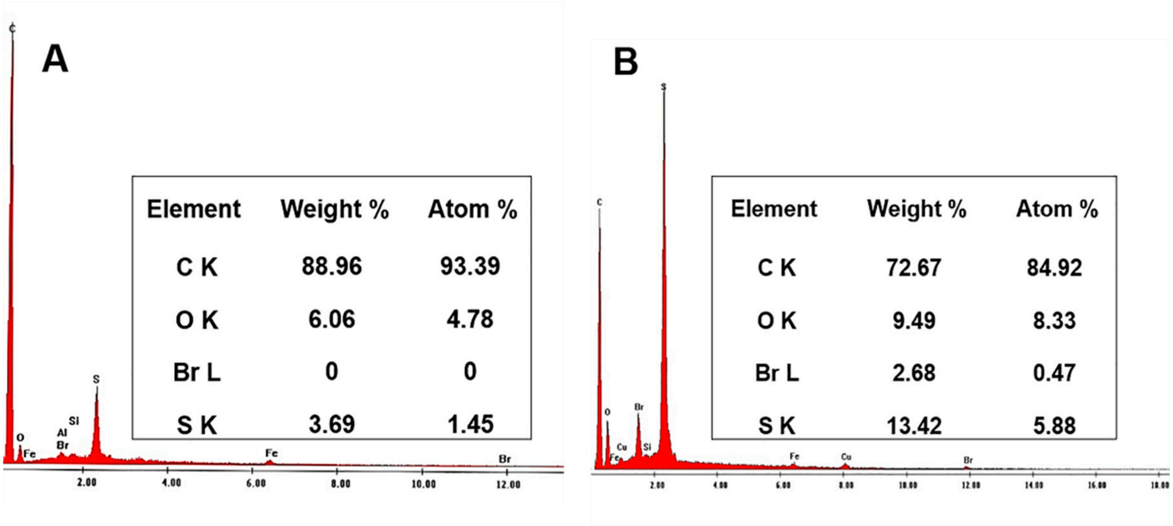

Figure S2. SEM-EDS analysis of bromine in A) SSEBS-PEDOT fibre mat and B) electropolymerized fibre mat bearing the brominated macroinitiator [SSEBS-PEDOT-P (EDOT-co-EDOTBr)].

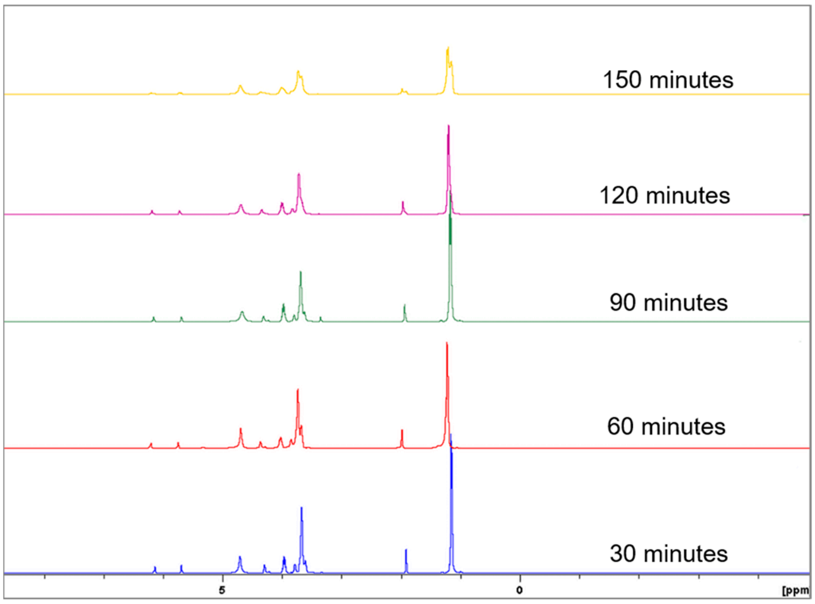

Figure S3. NMR spectra of OEGMA monomer from the ATRP reaction solution at different time intervals.

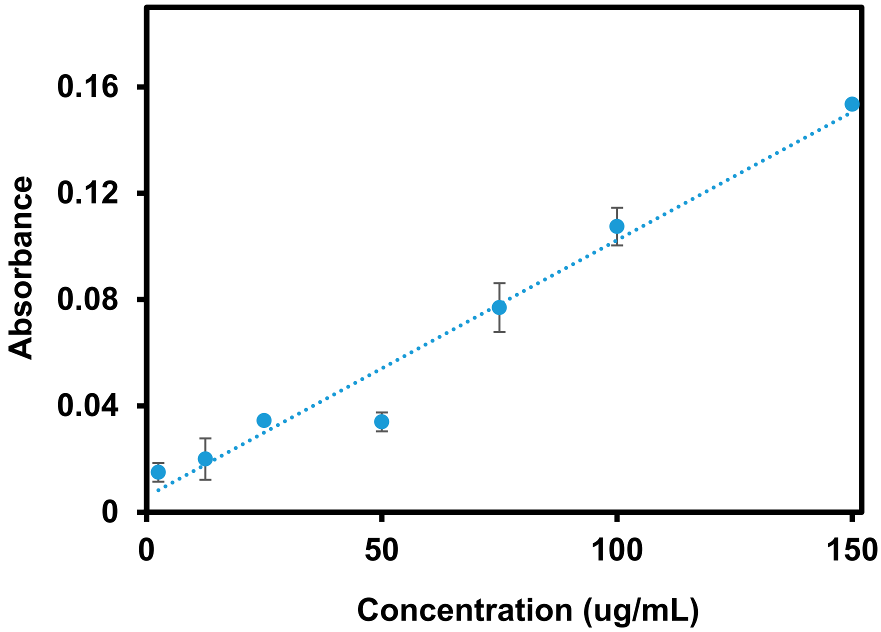

Figure S4. Calibration curve of BSA standards at different concentration vs absorbance measured using BCA protein assay.

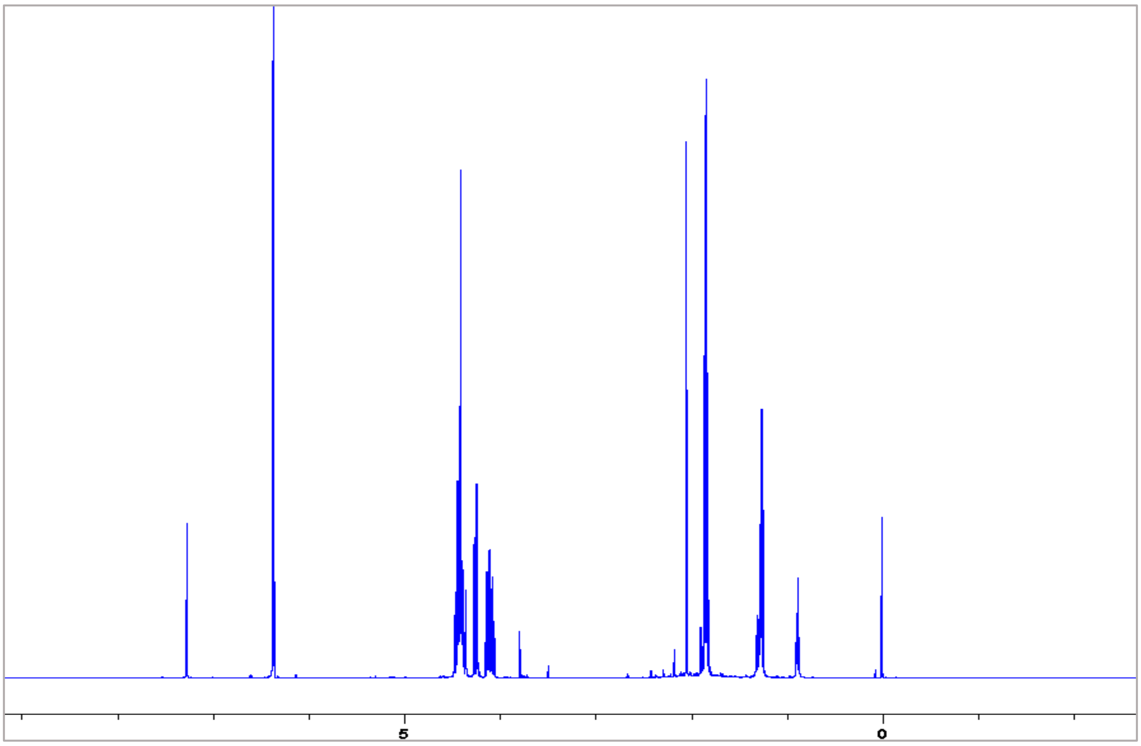

Figure S5. ^{1}H NMR spectrum of synthesized EDOTBr.

Table S1. Examples of antifouling modifications on different electrode surfaces.

Material	Modification	Results	Reference
Polymeric microsphere	Poly(sodium 4-styrenesulfonate) (PNaSS) crosslinked PEDOT microstructure - smart surface	The attached bacterial wall was destroyed by the application of constant potential >0.5 V.	[1]
Glassy carbon electrode	PEDOT/Hyaluronic acid (HA) electrodeposited on GCE	Changes in CV peak currents were very small due to excellent fouling resistance.	[2]
Glassy carbon electrode	PEDOT: PSS modification	Reduced fouling allows continuous monitoring of tricresyl phosphate.	[3]
Stainless steel substrate	PEDOT/Graphene oxide and PEDOT/PSS nanohybrid coating	PEDOT/GO and PEDOT/PSS showed excellent antifouling efficiency.	[4]
Glassy carbon electrode	PEDOT/Ionic liquid electrodeposited glassy carbon electrode surface	Ability to resist biofouling in complex human serum.	[5]
Electrospun fibre mat	sSEBS-PEDOT/P(EDOT-co-EDOTBr)-g-POEGMA brush grafted surface	>80 % antifouling efficiency	This work

References:

[1] Tomšík, E., Laishevkina, S., Svoboda, J., Gunar, K., Hromádková, J., and Shevchenko, N. (2022) Preparation of Smart Surfaces Based on PNaSS@ PEDOT Microspheres: Testing of E. coli Detection, *Sensors 22*, 2784.

[2] Wang, W., Cui, M., Song, Z., and Luo, X. (2016) An antifouling electrochemical immunosensor for carcinoembryonic antigen based on hyaluronic acid doped conducting polymer PEDOT, *RSC advances 6*, 88411-88416.

[3] Yang, X., Kirsch, J., Olsen, E. V., Fergus, J. W., and Simonian, A. L. (2013) Anti-fouling PEDOT: PSS modification on glassy carbon electrodes for continuous monitoring of tricresyl phosphate, *Sensors and Actuators B: Chemical 177*, 659-667.

[4] Hsu, C.-C., Cheng, Y.-W., Liu, C.-C., Peng, X.-Y., Yung, M.-C., and Liu, T.-Y. (2020) Anti-bacterial and anti-fouling capabilities of poly (3, 4-Ethylenedioxythiophene) derivative nanohybrid coatings on SUS316L stainless steel by electrochemical polymerization, *Polymers 12*, 1467.

[5] Song, Z., Sheng, G., Cui, Y., Li, M., Song, Z., Ding, C., and Luo, X. (2019) Low fouling electrochemical sensing in complex biological media by using the ionic liquid-doped conducting polymer PEDOT: application to voltammetric determination of dopamine, *Microchimica Acta 186*, 1-9.
